# The effect of short-term creatine intake on blood lactic acid and muscle fatigue measured by accelerometer-based tremor response to acute resistance exercise

**DOI:** 10.20463/pan.2020.0006

**Published:** 2020-03-31

**Authors:** Sinwook Lee, Gyuseog Hong, Wonil Park, Jaeseong Lee, Nahyun Kim, Hyejoon Park, Jonghoon Park

**Affiliations:** 1 Department of Physical Education, Korea University, Seoul Republic of Korea; 2 Convergence Center, LG Electronics, Seoul Republic of Korea

**Keywords:** creatine, supplement, muscle, fatigue, lactic acid, tremor, accelerometry, anaerobic exercise

## Abstract

**[Purpose]:**

The purpose of this study was to investigate the effects of short-term creatine intake on muscle fatigue induced by resistance exercise in healthy adolescent men, i.e., lactic acid concentration and wrist and head tremor measured by an accelerometer.

**[Methods]:**

Twelve healthy adolescent men who had no experience with creatine intake were included. The subjects were randomly assigned to the creatine group and the placebo group, followed by 5 days of creatine and placebo intake, and 5 times of 5 sets of leg press, leg extension, bench press, and arm curl exercises at 70% repetition maximum (RM). The lactic acid concentration before and after exercising, rate of perceived exertion (RPE), and accelerometer-based wrist tremor and head tremor during exercise were measured. Subsequently, after 7 days to allow for creatine washout, the same exercise treatment and measurement were performed in each group after switching drug and placebo between the groups.

**[Results]:**

The level of lactic acid before and after the acute resistance exercise trial was significantly lower in the creatine group than in the placebo group (P <0.05). The mean RPE during the resistance exercise was significantly lower in the creatine group than in the placebo group (P <0.05). There was no difference between the two groups in the mean wrist tremor during resistance exercise, but the mean head tremor values were significantly lower in the creatine group than in the placebo group in the arm curl, the last event of the exercise trials (P <0.05).

**[Conclusion]:**

Short-term creatine intake reduces the blood fatigue factor increased by resistance exercise, and is thought to suppress fatigue, especially in the latter half of resistance exercise. Therefore, these findings indicate that short-term creatine intake can have an improved effect on anaerobic exercise performance.

## INTRODUCTION

Systematic and scientific methods are constantly under exploration to improve exercise performance abilities. Nutritional supplements refer to substances that help increase the strength, speed, reaction time, or the endurance of an athlete. Additionally, they promote recovery after exercise, delaying the onset of fatigue in muscle fibers and neutralizing the inhibitory effect of the central nervous system on muscle contraction and other functions^1,2^.

Of late, creatine has been widely used as a nutritional supplement to improve anaerobic capacity^3^. Creatine (methylcarbamimidamido-acetic acid) is a nitrogenous amino acid, a natural product that can be taken through food. Since the amount required by the body can be satisfied by endogenous biosynthesis, it is not an essential nutrient although it is closely associated with the body's metabolic function^4^. More than 98% of creatine is found in the muscle where it takes the form of creatine phosphate (Pcr), a high energy phosphate compound produced by creatine kinase, and serves to store muscle contraction energy. In a study by Greenhaff et al.^5^, the intramuscular concentrations of Pcr increased after a daily total intake of 20 g creatinine (i.e., 5 g, 4 times a day) for 5 days. In particular, those with significantly lower creatine concentrations before the intake showed increased Pcr resynthesis during the recovery period^5^. In an experiment conducted on men, it was reported that the short-term creatine intake group with a daily intake of 20 g for 5 days showed an increase in the absolute total muscle strength, relative muscle strength, and the number of bench press repetition at 70% strength of 1 repetition maximum (RM)^6^. In another study with daily creatine intake of 20 g for 5 days in 14 healthy adult subjects, after performing 5 sets of bench press in 10RM up to the failure point and 5 sets of jump squat exercise at 30% strength of 1RM repeated 10 times, the creatine intake group showed an increase in the number of repetitions for the bench press exercise and a significant increase in the maximum power in the jump squat exercise when compared with placebo intake group^7^. Creatine intake is reported to affect the increase in the storage of Pcr in skeletal muscles, leading to a transient or temporary increase in the exercise performance and enhancement of the recovery between exercises^8^. As can be seen from these examples, creatine is thought to have a reducing effect on metabolic factors involved in fatigue when anaerobic exercise is performed.

One of the phenomena caused by metabolic fatigue, tremor, represents a variety of involuntary oscillations that occur in organisms^9,10^. Sakamoto et al.^11^ argued that physiological tremor refers to invisible mechanical vibrations of body parts, such as hands and fingers. As the muscle fatigue in the human body increases, the level of blood lactic acid concentration increases. Arihara and Sakamoto^12^ conducted a fatigue-inducing experiment to measure physiological tremor by hanging a weight from the middle finger. They reported that the amplitude of tremor increases with the progression of fatigue. When the muscles experienced fatigue, changes were noted in the time axis and frequency characteristics of the bio-signals^13^. The oscillations between 8 and 12 Hz generated by the effect of fatigue show an increase in peak output during muscle fatigue, and the tremor amplitude increases when the distal muscle of one arm experiences fatigue^14^. As a mechanism of tremor development, the causes of the regulation of contraction intensity occurring in the motor unit, peripheral stretch reflex loop, and central nervous system have been reported^15^.

Creatine levels are known to be high in the central nervous system^16^. Besides, creatine intake has been reported to have a possible positive effect on the regulation of contraction intensity in the central nervous system^17,18^. In this study, it was hypothesized that creatine intake would reduce tremor during resistance exercise not only by reducing fatigue in the peripheral tissues through lactic acid reduction, but also through the positive effects of decreasing central nervous system fatigue. Although the positive effects of creatine intake on muscle fatigue during resistance exercise have been reported in many previous studies, to date, the effect of creatine intake on inhibition of tremor induced by resistance exercise has not been reported. By measuring the lactic acid concentration and wrist and head tremor with an accelerometer, we aimed to investigate the effects of shortterm creatine intake on muscle fatigue induced by resistance exercise in healthy adolescent men.

## METHODS

### Subjects

The subjects included 12 healthy adolescent men aged between 17 and 20 attending a high school in Y city, Gyeonggi-do (Table 1). After explaining the purpose and schedule of the study, subjects who voluntarily decided to participate signed the informed consent form while their legal representatives gave written consent approving participation. All included subjects met the following three conditions; no specific disease, no experience of resistance exercise in the past year, and no history of specific medications or creatine intake for at least 6 months before the start of the study. The subjects were divided into two groups by random assignment. This study was conducted with the approval of the Institutional Review Board of K- university (KUIRB-2019-0338-01).

**Table 1.** Characteristics of research participants.

Variables	
Subject (n)	12
Age (years)	18.92 ± 0.51
Height (cm)	174.24 ± 5.04
Weight (kg)	70.58 ± 9.01
BMI (kg/m^2^)	23.19 ± 2.37
Percent body fat (%)	14.74 ± 4.42
Body fat mass (kg)	10.61 ± 4.17
Waist-Hip Ratio	0.82±0.03
Systolic blood pressure (mmHg)	122.50 ± 4.01
Diastolic blood pressure (mmHg)	69.25 ± 7.05

### Study procedure

The study subjects were placed into two groups (Ncr, the non-creatine intake group, and Cr, the creatine intake group). After the subjects were completely familiarized with the study objectives and measurement methods, their height and weight were measured, body composition tests conducted, and 1RM of experimental items, such as leg press, leg extension, bench press, and arm curl was measured. As a preliminary experiment, after 3 days of rest, both groups (groups A and B) performed 5 times of 5 sets of leg press, leg extension, bench press, and arm curl exercises at 70% strength of 1RM without creatine or placebo intake; lactic acid concentration before and after exercise, and RPE and tremor during exercise were measured. After 7 days of washout, group A had a daily creatine intake of 20 g (10 g × 2 times) while group B had placebo intake for 5 days followed by the first measurement on day 6.

Measurements were done in the afternoon after fasting for 4 hours after breakfast. After wearing a wireless heart rate monitor, the subjects were allowed to lie down on a mat in the lab and allowed to rest for 30 minutes. After stabilization, the heart rate was recorded, lactic acid and blood pressure values measured before the experiment, and a survey on hours of sleep conducted after the measurement. Before measurements and with an accelerometer worn on the subjects’ wrists and ears, leg press and extension, bench press, and arm curl experiments were performed. During the experiment, heart rate, and perceived exertion were measured for each set, and lactic acid concentration was measured immediately after exercise. After 7 days of washout (creatine excretion), group A had placebo while group B had daily creatine of 20 g (i.e., 10 g × 2 times per day) for 5 days each. The second experimental measurements were done on day 6. The experimental procedure and method were identical to the first experiment.

### Exercise trials

To prevent sustaining an injury from the resistance exercise, warm-up exercises in the form of warm-up and cooldown stretching were performed for 10 minutes before and after exercise intervention, respectively. Before measuring the 1RM of the leg press, leg extension, bench press, and arm curl, the study assistant demonstrated different postures for each exercise. The exercise equipment used in the study was Vr3 leg press (Cybex, USA), Vr3 leg extension (Cybex, USA), chest press (Infinity, Korea), and arm curl (Infinity, Korea). Synthesized creatine is mainly stored in skeletal muscles, of which about 60% is in the form of phosphocreatine (Pcr), which produces and supplies ATP through a creatine-phosphate shuttle during short-time anaerobic exercise [19]. Therefore, the exercise intervention was slightly modified from those done in previous studies^6,20^. The exercise consisted of 5 sets for each event, 5 times for each set at 70% RM strength, which tends to consume much creatine in a short time. A 1-minute break was taken after each set and a 3-minute break when switching to the next exercise.

1) During a leg press exercise with 70% strength of 1RM, the subject sat on a leg press bench, adjusted the length of the backrest according to his height, placing both feet together at a distance 2/3rd from the footrest, and with the hands placed on the side handles. One round of exercise was considered to start from the point when the legs were extended, to the end when the legs were extended again. By pressing on the footrest, the legs were extended during the muscle contraction and carefully touched the rod positioned between the weight bars during the muscle relaxation and this was repeated 5 times for a total of 5 sets.
2) During the leg extension exercise with 70% strength of 1RM, the subject adjusted the length of the backrest and the length of the leg according to his height and placed his hands on the side handles. One round of the exercise was considered to start from the point when the legs were bent, and to end when the legs were bent again. The legs were extended to the knee height during the muscle contraction and carefully touched the rod positioned between the weight bars during the muscle relaxation and this was repeated 5 times for a total of 5 sets.
3) During bench press exercise at 70% strength of 1RM, the subject laid on the bench and adjusted the height according to a fixed height lever, so that the hands were placed wider than the shoulder width while the entire soles of both feet were firm on the ground. One round of exercise was considered to start from the point when the arms were extended, and to end when the arms were extended again. The arms were extended, with the strength provided by the pectoral muscle during muscle contraction, and carefully used to touched the rod positioned between the weight bars during muscle relaxation and this was repeated 5 times for a total of 5 sets.
4) During arm curl exercise at 70% strength of 1RM, the subjects were seated on the bench and the height adjusted accordingly such that the arms made contact with the bar at about the shoulder width. The bar was contracted as much as possible during the muscle contraction phase such that it carefully touched the rod positioned between the weight bars during the muscle relaxation and this was repeated 5 times for a total of 5 sets. Additionally, during the 4 resistance exercises, the subjects were guided to maintain proper breathing methods and postures, and enough practice was performed before the exercises were conducted.

### Creatine intake

In this study, monohydrate creatine (Nutricost, USA) was used. Twelve subjects were divided by random assignment into two groups to perform a single-blinded experiment. During the experiment period, the usual diet was maintained but other types of medication intake and administration were prohibited. Subjects were instructed to maintain the same pattern with regards to meals and lifestyle as much as possible. For placebo treatment, corn starch (Corn Starch, Korea), which was the same color as creatine and which could not be distinguished by appearance or taste when mixed with lemonade (No brand Co. Ltd., Korea), was used. Twenty grams of creatine or placebo were mixed in 250 ml of lemonade and served at the same time. The total amount of creatine was 100 g of which 20 g per day was administered orally for 5 days followed by experimentation on the 6th day of the study.

### Body composition

Height was measured using an automatic anthropometer (SH-9600A Sewoo System, Korea) and was done with the subjects wearing comfortable clothes, and stepping on the footrest barefoot with the body straight and in close contact with the equipment. Body weight, skeletal muscle mass, lean body mass, body fat mass, and body fat percentage were analyzed with a body fat analyzer (Inbody 220, BioSpace, Korea) based on the electric resistance principle.

### 1RM test

The exercise intensity was set using 1RM, which refers to the muscle's ability to lift to the maximum at one time; this indicates the maximum muscle strength that can be exerted against a heavy weight. For example, it is to find a weight that can be repeated once with all the force during a bench press. The 1RM measurement was based on the 1RM indirect estimation method^21^.

·1RM indirect estimation formula 1RM = Wo + WI
·Wo = Weight considered to be repeatable 7 - 8 times after warming-up
·W1 = Wo x 0.025 x R (R= actual number of repetitions)

### Rating of Perceived Exertion (RPE)

The steps were explained before the experiment. In the 15-point Borg scale, a 6-point scale indicates requiring no effort at all^22^, and a 19-point scale represents an extremely exhausted condition. After each set, subjects were asked to indicate the applicable exertion scale

### Blood lactic acid concentration

Fingertips were used to measure lactic acid concentration using the Lactate pro 2 (Arkray. Japan) lactic acid analyzer. The blood lactic acid concentration was measured during rest before the experiment and immediately after exercise

### Tremor

Tremor: Tremor data using an accelerometer was obtained through sensors attached to the wrists and to the ears, which are mainly involved in the experimental postures. An accelerometer (Accelerometers, LG, Korea) was attached to the radius and left ear and the axis of the sensor was aligned so that it was perpendicular to the flat surface. The accelerometer was calibrated by adjusting the zero point in DC mode and tested in AC mode, and data were acquired during exercise with the accelerometer in place. The tremor waveform data during exercise were transformed into numbers through filtering, and the amplitude of the tremor was calculated from the mean value as the root mean square (RMS) value^23^.

### Heart rate

Heart rate was measured using a wireless heart rate monitor (Polar, s610i, Finland). The transmitter, which is a device that detects the heart rate signal and transmits it to the monitor via Bluetooth, is placed at the center of the subject's chest, with both arms wide open, and a belt is worn along the perimeter. The watch with the heart rate displayed was worn by the assistant to check the heart rate. Measurements were made before exercise to record the resting heart rate. The heart rate variation between sets was measured during exercise

### Blood pressure

Blood pressure was measured using a blood pressure monitor (Omron, HEM-780, Japan) and systolic and diastolic pressures were measured at rest before exercise

## STATISTICS

All statistical analyses were performed using SPSS Ver. 24.0 (SPSS Inc., Chicago, USA). The statistical significance level was set to P <0.05. As for the variables of the group, the mean (standard) and standard deviation (SD) of the descriptive statistics were calculated through descriptive statistical analyses to compare the results with and without creatine, and before and after the experiment. During the resistance exercise, the Bonferroni post hoc test was performed to compare and verify the heart rate and perceived exertion between groups. A paired t-test was conducted to compare the two groups on the hours of sleep, the rate of increase of lactic acid concentration before and after exercise, heart rate during exercise, perceived exertion, and wrist and head tremor.

## RESULT

The basic data including age, body composition, and blood pressure of the study subjects are shown in Table 1. With regards to hours of sleep on the day before exercise intervention, which significantly affects fatigue during exercise, there was no significant difference between the two groups in the mean hours of sleep before the experimental intervention, with and without creatine intake (Ncr: 7.08 ± 0.90 hr; Cr: 7.08 ± .90 hr, p = 0.870).

**Figure 1.**
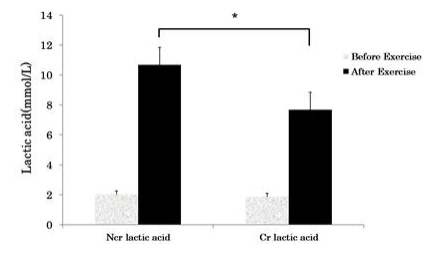
Blood lactic acid concentration before and after resistance exercise trial. Ncr: Non-Creatine intake group; Cr: Creatine intake group. *Significant difference between groups (*p* <0.05).

Blood lactic acid concentrations before and after the resistance exercise are shown in Figure 1. Blood lactic acid concentrations of the Ncr group and Cr groups before exercise were 2.04 ± .024 and 1.89 ± .23 mmol/L, respectively, and the concentrations in the Ncr group and Cr group after exercise were 10.69 ± 1.81 and 7.69 ± 1.16 mmol/L, respectively. The blood lactic acid concentration after exercise intervention was significantly lower in the Cr group than in the Ncr group (p <0.05).

**Figure 2.**
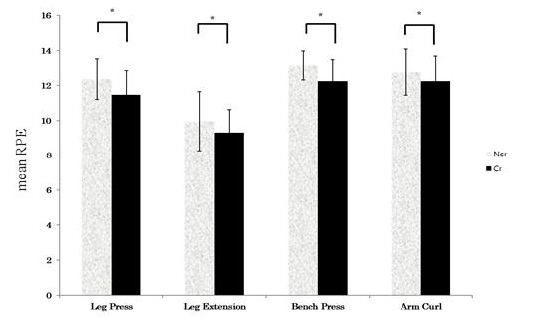
Mean Rating of Perceived Exertion (RPE ) during four different types of resistance exercises. Ncr: Non-Creatine intake group; Cr: Creatine intake group. *Significant difference between groups (*p* <0.05).

The mean RPE values during the resistance exercises for each exercise type were as follows: leg press (Ncr, 12.37 ± 1.17; Cr, 11.48 ± 1.37), leg extension (Ncr, 9.95 ± 1.71; Cr, 9.32 ± 1.30), bench press (Ncr, 13.15 ± 0.84; Cr, 12.25 ± 1.24) and arm curl (Ncr, 12.77 ± 1.32; Cr, 12.25 ± 1.43), indicating that the Cr group showed significantly lower RPE values than the Ncr groups in all four types of exercises (p <0.05) (Figure 2).

**Figure 3.**
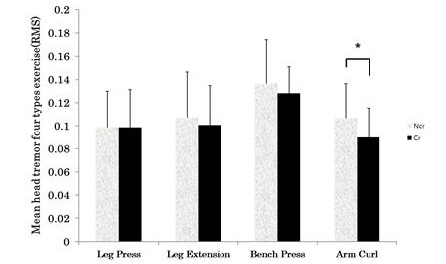
Mean head tremor during four different types of resistance exercises. Ncr: Non-Creatine intake group; Cr: Creatine intake group. *Significant difference between groups (*p* <0.05).

During the resistance exercise, the mean RMS value of the head tremors were as follows: leg press (Ncr, 0.0988 ± 0.031; Cr, 0.0988 ± 0.032), leg extension (Ncr, 0.1074 ± 0.039; Cr, 0.1007 ± 0.034), bench press (Ncr, 0.1365 ± 0.011; Cr, 0.1283 ± 0.006), and arm curl (Ncr, 0.1069 ± 0.029; Cr, 0.0900 ± 0.025); during the arm curl exercise - the last type in the order of exercises, the Cr group showed a significantly lower RMS value than the Ncr group. (p <0.05) (Figure 3).

**Figure 4.**
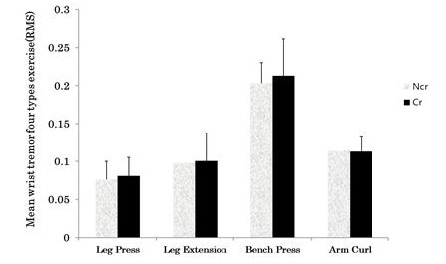
Mean wrist tremor during four different types of resistance exercises. Ncr: Non-Creatine intake group; Cr: Creatine intake group.

The mean RMS values of the wrist tremors during resistance exercise were as follows: leg press (Ncr, 0.0764 ± 0.024; Cr, 0.810 ± 0.024), leg extension (Ncr, 0.0983 ± 0.027; Cr, 0.1005 ± 0.037), bench press (Ncr, 0.2032 ± 0.036; Cr, 0.2131 ± 0.048), and arm curl (Ncr, 0.1144 ± 0.019; Cr, 0.1129 ± 0.019). After analyzing wrist tremors for each of the four types of resistance exercise, no significant difference was found between the two groups. (Figure 4).

## DISCUSSION

The main results of this study are as follows: short-term creatine intake suppressed the increase in blood lactic acid after resistance exercise. Besides, the RPE measured during resistance exercises was significantly lowered by creatine intake. The effect of creatine intake on wrist tremors was not observed during exercise, but the head tremor level was suppressed by creatine intake in the arm curl, the last part of the exercise sequence. Therefore, it can be said that short-term creatine intake is effective in suppressing muscle fatigue.

The total time per set of exercises performed in this study did not exceed 30 seconds per set for all events. It is said that high-intensity exercise increases the use of the fast-twitch fiber (Type Ⅱ fiber) within 30 seconds and energy is obtained through the adenosine triphosphate and phosphocreatine (ATP-PC) system or glycolysis corresponding to the resynthesis of anaerobic energy^24^. In general, the use of fast-twitch fiber is known to increase the production rate of lactic acid due to high fatigue induction^25,26^. A study on oral administration of creatine to rugby players^27^ reported that creatine intake for 5 days of 20 g per day before resistance exercise decreased lactic acid levels after resistance exercise (placebo group, 9.64 ± 1.78; creatine group, 8.36 ± 1.64 mmol/L). In the study by B.S. Heo and J.G. Ji^28^, oral administration of creatine to boat racers showed that daily intake of 20 g of creatine intake for 4 days significantly decreased lactic acid levels after resistance exercise (placebo group, 9.00 ± 0.55; creatine group, 6.88 ± 0.46 mmol/L). As in the previous studies, this study also showed that lactic acid levels after resistance exercise significantly decreased after creatine intake (placebo group, 10.69 ± 1.81; creatine group, 7.69 ± 1.16 mmol/L). Creatine increases the resynthesis process of Pcr and suppresses H⁺ formation, the cause of fatigue induction in the process of ATP production, and helps increase exercise performance as an acidifying buffer in the active muscle during exercise^29,30^. Also, an increase in the amount of creatine stored in the body through intake improves the mobilization rate for anaerobic energy resynthesis, thereby causing an increase in energy supply by glycolysis. This decreases anaerobic glycogen metabolism and affects the Ca2⁺-ATP activity by reducing the relaxation time after muscle contraction and increasing the cross-bridging rate of actin and myosin^31-35^. Therefore, it was confirmed in this study that short-term creatine intake effectively reduced the production of lactic acid during high-intensity resistance exercise.

With the resistance exercise composed of several sets, RPE gradually increases as muscle fatigue increases and as the exercise progresses along with these sets^36^. As blood lactic acid accumulates, perceived exertion increases during resistance exercise of complex joint exercise protocols; increased blood lactic acid is also associated with muscle damage and discomfort^36,37^. The RPE is used as a physiological indicator of muscle fatigue along with blood lactic acid.^20,38^. In this study, as a result of conducting 5 sets × 5 times of leg press, leg extension, bench press, and arm curl resistance exercise at 70% strength level of 1RM, the RPE decreased with creatine intake. Suminski et al.^20^ reported a significant positive correlation between RPE by resistance exercise at 70% strength of 1RM and blood lactic acid concentration. Therefore, the decrease in RPE in this study is thought to have occurred through creatine intake contributing to the decrease in lactic acid concentration, a blood fatigue substance after resistance exercise; decrease in lactic acid leading to decrease in muscle fatigue and lowering of the RPE.

Most of the previous studies on tremors have been reported in relation to muscle fatigue. The amplitude of tremor varies depending on the external load; and muscle fatigue caused by irregular increases in muscle strength is the main cause of the increased amplitude of tremor as it decreases muscle activity^39,35^. The change in tremor amplitude and frequency is determined by the degree of fatigue and its duration^39,40^. Limb tremor was found to be increased through heat generation and exercise-induced fatigue after exercise^14,41^. Petrofsky et al.^42^ reported that if muscle contraction persists, the value of electromyography in integral expression, which represents muscle fatigue, increases at a similar rate along with muscle strength. According to a recent meta-analysis of 63 studies, creatine supplementation showed a beneficial effect in squat and leg presses with an increase of 8% and 3% in RM, respectively^43^. In this study, as a result of analyzing the resistance exercise for each type of exercise, there were no significant differences in the head tremor during leg press, leg extension, and bench press; but the head tremor was lowered by creatine intake in the arm curl, the last item of the exercise intervention. Creatine helps to maintain constant strength by mobilizing new exercise units when the exercise units mobilized during the resistance suppression stops due to fatigue in the second half of the exercise^44^. In addition, creatine is known to decrease blood fatigue factors, such as lactic acid by promoting the resynthesis of Pcr, and as a result, affects the decrease in tremor. Another recent study also showed that creatine intake has a positive effect on restoring physical and mental performance after sleep deprivation, and in particular, has a neuroprotective effect when cellular energy supply is impaired, as in the case of severe oxygen starvation^45-47^.

In this study, wrist tremor increased with the resistance exercise during leg press, leg extension, bench press, and arm curl exercises at 70% strength of 1RM, but the effect of creatine intake was not observed. It is considered that since a fixed type machine was used, there was a limitation in the wrist tremor, whereas head was not fixed, thus the body tremor from the muscle fatigue was transferred to the accelerometer attached to the ear without problems. In this study, a fixed-type exercise machine was used, not dumbbells, as an exercise trial device for lower and upper body exercise intervention to exclude external factors, such as direction and angle. There may have been a limit to measuring wrist tremor during upper body exercise, because during the resistance exercise, the subject held the bar connected to the weight bar and the bar that supported the body in the upper body exercise and the lower body exercise, respectively.

The limitations of this study are as follow: first, we cannot rule out the possibility that various factors, such as movements and angle affected the measurement of tremor during exercise. In this study, we tried to exclude these external factors as much as possible by using the fixed-type machine and time control. In future studies, the effect on wrist tremor should be measured at the isometric contraction state before and after exercise. Second, this study population was made up of only adolescent men; thus, the results of this study cannot be applied equally to women. Therefore, future studies on female subjects or the elderly are warranted.

To conclude, this study investigated the effect of short-term creatine intake under fatigue-inducing conditions caused by repeating sets of exercise types during resistance exercise. This is the first study to report a change in tremor as the fatigue index with creatine intake during resistance exercise. In this study, lactic acid and RPE levels after resistance exercise were reduced through short-term creatine intake, and the results also showed a decrease in head tremor in the latter phase of exercise. These results indicate that short-term creatine intake may improve anaerobic exercise performance.

